# Membrane damage mechanism contributes to inhibition of trans-cinnamaldehyde on *Penicillium italicum* using Surface-Enhanced Raman Spectroscopy (SERS)

**DOI:** 10.1038/s41598-018-36989-7

**Published:** 2019-01-24

**Authors:** Fei Huang, Jie Kong, Jian Ju, Ying Zhang, Yahui Guo, Yuliang Cheng, He Qian, Yunfei Xie, Weirong Yao

**Affiliations:** 1 State Key Laboratory of Food Science and Technology, Jiangnan University, Jiangsu Province, China; 2 School of Food Science and Technology, Jiangnan University, Jiangsu Province, China; 3 Joint International Research Laboratory of Food Safety, Jiangnan University, No. 1800 Lihu Avenue, Wuxi, 214122 Jiangsu Province China

## Abstract

The antifungal mechanism of essential oils against fungi remains in the shallow study. In this paper, antifungal mechanism of trans-cinnamaldehyde against *Penicillium italicum* was explored. Trans-cinnamaldehyde exhibited strong mycelial growth inhibition against *Penicillium italicum*, with minimum inhibitory concentration of 0.313 μg/mL. Conventional analytical tests showed that trans-cinnamaldehyde changed the cell membrane permeability, which led to the leakage of some materials. Meanwhile, the membrane integrity and cell wall integrity also changed. Surface-enhanced Raman spectroscopy, an ultrasensitive and fingerprint method, was served as a bran-new method to study the antifungal mechanism. Characteristic peaks of supernatant obviously changed at 734, 1244, 1330, 1338 and 1466 cm^−1^. The Raman intensity represented a strong correlation with results from conventional methods, which made SERS an alternative to study antifungal process. All evidences implied that trans-cinnamaldehyde exerts its antifungal capacity against *Penicillium italicum* via membrane damage mechanism.

## Introduction

China is a large producer of citrus fruits^1^, quantities of citrus fruits are stored for a long time for sale. Blue mold caused by *Penicillium italicum* (*P. italicum*) is one of the main diseases of citrus fruits during storage^2^. Postharvest decay leads to tremendous economic losses^3^. In-depth study of the inhibitory mechanism of fungistat against *P. italicum* will help to develop more efficient and more reasonable new fungistat.

The development of isolates resistant to traditional synthetic chemicals involved in human health and environmental pollution has urged scientists to find new approaches to control the pathogens. Essential oils are new perspective to control citrus postharvest spoilage fungi, eliciting low toxic effects on mammals and strong inhibitory effects on food- or crop-contaminating fungi, such as *P. digitatum* and *P. italicum*^4^. Trans-cinnamaldehyde, main component of cinnamon oil and a safe food additive widely used in food industry^5^, exerts an excellent growth inhibition of bacteria and fungi^6,7^. However, antifungal mechanism of essential oils against fungi remains in shallow study, and molecular level research was rarely reported. Traditional characterization methods do not provide fingerprint analysis at molecular level.

Raman spectroscopy is a vibrational spectroscopic method that provides the fingerprint characteristics on various chemical and biochemical components in a complex system^8,9^. But Raman signal is relatively weak^10^, which limited its applications. Surface-enhanced Raman spectroscopy possesses extremely high sensitivity and provide fingerprint information of various molecules with the strong electromagnetic enhancement typically provided by Ag or Au NPs, the enhancement reaches 10^3^–10^6^ fold compared with the normal Raman scattering process^11^, which makes SERS an ultrasensitive detection tool, even down to single molecule level^12^. Thus, the changes in cellular composition such as in lipids, proteins, and nucleic acids can be monitored by SERS^13^. Due to its advantages, many researches were conducted with SERS to study the antibacterial activity and mechanism of drugs^14–16^ or particles^13,17^. However, utilize of SERS to research the antifungal activity and mechanism of essential oils was rarely reported. Only few literature had recorded the brilliant study of antifungal mechanism using SERS, nevertheless, they were just limited to studying yeast^18–20^. Thus, SERS seems to be a new and potential alternative for antifungal mechanism studying.

In this research, antifungal mechanism of trans-cinnamaldehyde on *P. italicum* was going to be studied with the traditional methods, such as antifungal assay, test of permeability of cell membrane, and determination of integrity of plasma membrane and so on. Sequentially, SERS was planned to be developed as a new alternative to analyze the antifungal mechanism. After preparing colloidal Au nanoparticles, the supernatant of trans-cinnamaldehyde treated mycelium suspension would be detected using SERS. Some typical strong peaks was likely to be assigned to nucleic acid, protein and lipid. The correlation between Raman intensities and the results obtained from traditional methods could be analyzed. This report promise the potential application of SERS in interpretation of the antifungal process in future.

## Material and Methods

### Chemicals and pathogen

Trans-cinnamaldehyde (≥95%) was purchased from Shanghai Shuangxiang auxiliaries plant (Shanghai, China). Propidium iodide (≥95%), Bovine serum albumin (≥96%), potassium auric chloride (original Au ≥99.95%), and sodium citrate (99%) were bought from Nanjing Senbeijia biological technology Co., Ltd (Nanjing, China). Coomassie brilliant blue G-250 (90%) was obtained from Sinopharm chemical reagent Co., Ltd (Shanghai, China). All the chemicals were analytic grade. The water, except special statement, was deionized water.

*P. italicum* was obtained from China General Microbiological Culture Collection Center (CGMCC) (Beijing, China). All the test strains were preserved on potato dextrose agar (PDA) at 28 ± 2 °C. Conidial spore concentration was adjusted to 1 × 10^6^ cfu/mL with Haemocytometer.

### Antifungal assay

Several essential oils were tested to assess their capacity of inhibitory effect against *P. italilum*. The experiment was demonstrated using Microtitre Plate Well Assay^21^. 100 μL sterilized potato dextrose broth (PDB) was injected into 96-well plates. 100 μL essential oils diluted in ethanol was injected into the first well, 2 fold diluted. Then, 100 μL conidial spore suspension (1 × 10^6^ cfu/mL) was poured into each well. Finally, the 96-well plate was incubated at 28 ± 2 °C in the constant temperature incubator for 48 h. The lowest concentration that completely inhibited the growth of the fungus was considered the minimum inhibitory concentration (MIC).

The mycelial growth inhibition was carried out following previous work^22^. Different amounts of trans-cinnamaldehyde were added to 50 mL PDB liquid medium with the final concentration of trans-cinnamaldehyde 0.000, 0.020, 0.040, 0.078, 0.156, 0.313 0.625 μL/mL. Then, 200 μL conidial spore suspension was inoculated in each triangle bottle, which was incubated at 28 ± 2 °C in a rotatory shaker at 120 r/min for 2 d. Fungal growth inhibition was estimated gravimetrically by weighting the biomasses after drying at 80 °C to a constant weight. The percentage of mycelial growth inhibition (PGI) was calculated according to the following formula:

$${\rm{PGI}}( \% )=[\frac{wc-wt}{wc}]\times 100$$

where *wc* (g) is net dry weight of control fungi and *wt* (g) is net dry weight of treated fungi.

### Test of plasma membrane integrity

At the beginning, aliquot of 1 mL conidial spore suspension was transferred into 50 mL triangle bottles with 10 mL PDB to obtain a final concentration of 1 × 10^6^ cfu/mL, incubated at 28 ± 2 °C in a rotatory shaker at 120 r/min for 7 h. Different amounts of trans-cinnamaldehyde were added to triangle bottles. PDB without trans-cinnamaldehyde served as control. To assay the effect of trans-cinnamaldehyde on plasma membrane integrity, conidial spores of *P. italicum* were collected from PDB medium at 0, 2, 4 and 6 h.

Membrane integrity was assayed following reported work^23^ with some modifications. The time of collecting conidial spores was described above. Spores in PDB were collected by centrifugation at 10000 × g for 3 min at room temperature, and washed twice with 0.2 mol/L sodium phosphate buffer (pH 7.0) to remove residual medium. The conidial spores were stained with 10 μg/mL propidium iodide (PI) for 5 min at 30 °C. Spores were then collected by centrifugation, washed twice with the sodium phosphate buffer (pH 7.0) to remove residual dye. The spores were observed with Inverted Fluorescence Microscope (Carl Zeiss Vision, Germany) equipped with an individual fluorescein rhodamine filter set (Zeiss no. 15: excitation BP 546/12 nm, LP590 nm). Five fields of view from each cover slip were chosen randomly, and the number of spores in bright-field was defined as the total number. Membrane integrity (MI) was calculated according to the formula:

$${\rm{MI}}( \% )=\frac{NO.\,of\,total\,spores-No.\,of\,stained\,spores}{No.\,of\,total\,spoes}\times 100$$

### Measurement of reactive oxygen species

Determination of accumulation of reactive oxygen species (ROS) was carried out by reference to luminol chemiluminescence. Two-day-old mycelium from PDB collected by vacuum filtration was grounded into powder with liquid nitrogen. The supernatant from centrifuge was put on ice waiting for next step. 50 μL supernatant and 830 μL HEPES buffer (0.02 mol/L, pH 7.4) were injected into a glass container. Start MPI-B Chemiluminescence analysis test system (Xi’an Ruimai Analytical Instrument Co., Ltd., China) immediately when 20 μL horseradish peroxidase (3 mg/mL) was added under absolutely dark environment.

### Measurement of intracellular constituents leakage

#### Measurement of soluble protein leakage

Measurement of intracellular soluble protein leakage was accorded to the method reported previously^24^ with modifications. An aliquot of 200 μL conidial spore suspension (1 × 10^6^ cfu/mL) was transferred to triangle bottles with 100 mL PDB, cultured at 28 ± 2 °C in a rotatory shaker at 120 r/min for 2 d. Mycelium was collected by vacuum filtration, washed twice with sterilized distilled water. About 1 g fresh mycelium was resuspended in 30 ml 0.85% saline, into wich trans-cinnamaldehyde was poured and adjusted to 0, 0.5, 1, 2 MIC. The bottles were inoculated at 28 ± 2 °C in a rotatory shaker at 120 r/min. 2 mL of the suspension was collected at 0, 30, 60, 120 min, and centrifuged at 10000 × g, 5 min. 1 mL of supernatant was transferred into Eppendorf tube and 5 mL of 0.1 mg/mL Coomassie brilliant blue G-250 followed. After 10 min inoculated at room temperature, absorbance at 595 nm was measured. Bovine Serum Albumin (BSA) served as standard. The results were expressed as μg of soluble protein per g of fresh mycelium. The experiment was conducted three times.

#### Measurement of release of constituents absorbing at 260 nm

The release of constituents absorbing at 260 nm was measured referring to reported method^25^ with minor modifications. About 1 g fresh mycelium was resuspended in 50 mL 0.85% saline, into wich trans-cinnamaldehyde was poured and adjusted to 0, 0.5, 1, 2 MIC. The bottles were inoculated at 28 ± 2 °C in a rotatory shaker at 120 r/min. 2 mL of the suspension was collected at 0, 30, 60, 120 min, and centrifuged at 10000 × g, 5 min. 1 mL of supernatant was used to determine the constituents absorbing at 260 nm by absorbance at 260 nm.

### Determination of potassium ions efflux

A previously reported method was used to determine the amount of potassium ions efflux^26^ with modifications. Detailedly, two-day-old mycelium cultured in PDB was collected by vacuum filtration. The concentration of free potassium ions in suspension was determined after the exposure to trans-cinnamaldehyde at 0, 0.5, 1, 2 MIC in ultrapure water for 0, 30, 60, 120 min. At each pre-established interval, free potassium ions in the supernatant was measured by a photometric procedure using the flame atomic absorption spectroscopy (Varian, USA). Results were expressed as amount of extracellular free potassium (mg/L) in the growth medium in each interval of incubation.

### SEM of mycelium structure after treatment

Mycelium during logarithmic phase was pre-fixed with 5% glutaraldehyde in 0.1 mol/L phosphate buffer, pH 7.2, and rinsed with 0.1 mol/L phosphate buffer. Then the mycelium was pro-fixed with 1% tannic acid in 0.1 mol/L phosphate buffer, pH 7.2, and rinsed with 0.1 mol/L phosphate buffer, following which they were dehydrated in an ethanol series (30%, 50%, 70%, and 90%, v/v). After drying at the critical point in liquid carbon dioxide, the sample was attached to a sample stage. The specimen were coated with gold in an BAL-TEC, SCD 005 ion sputter (Hitachi, Japan), and then a FEI-Quanta 200 scanning electron microscope (Hitachi, Japan) was used to observe the sample, operating at 10 kV at 1200x level of magnification.

### Preparation of colloidal Au nanoparticles for SERS

6 mL 1% potassium auric chloride was added to the round-bottom flask, 94 mL ultrapure water followed. The round-bottom flask was heated in oil bath at 120 °C. After ebullition, 4 mL sodium citrate was poured into the system. After maintaining ebullition for 30 min, the colloidal Au nanoparticles was prepared, and preserved in a brown bottle. All the containers were immersed in aqua regia overnight to wash impurities away.

### SERS measurement of extracellular material

About 1 g two-day-old mycelium was collected with vacuum filtration and resuspended in 50 mL triangle bottle containing 20 mL 0.85% saline where trans-cinnamaldehyde was at 0, 0.5, 1, 2 MIC, respectively. 1 mL suspension was transferred to Eppendorf tube and centrifuged at 10000 × g, 5 min. 100 μL supernatant was drop in a glass cup (0.5 cm diameter) containing 400 μL colloidal Au nanoparticles, and mixed them thoroughly, then SERS was tested by the Raman spectrometer (OptoTrace Technologies, Inc. California, USA) with a fiber-optic probe and a CCD array detector. The emitted wavelength was 785 nm and the output power was 285 mW. Tree or more spectrum were gathered from one sample on different spots in order to ensure the reproducibility of SERS features.

### Statistical analysis

All tests were conducted in triplicate, and all parameters were expressed as mean ± SD. Analysis of variance using one-way ANOVA followed by Duncan’s test was performed to test the significance of differences between means obtained among the treatments at the 5% level of significance using SPSS statistical software package release 22.0 (SPSS Inc., Chicago, IL, USA). The SERS spectrum data were processed by originPro 8.0 (OriginLab Corporation, USA), and the trend of Raman intensity elaborated in figures were expressed as the intensity of every peak using the baseline and peak function of OriginPro 8.0 (OriginLab Corporation, USA).

## Results and Discussion

### Inhibitory effect of trans-cinnamaldehyde on mycelial growth

**Figure 1:**
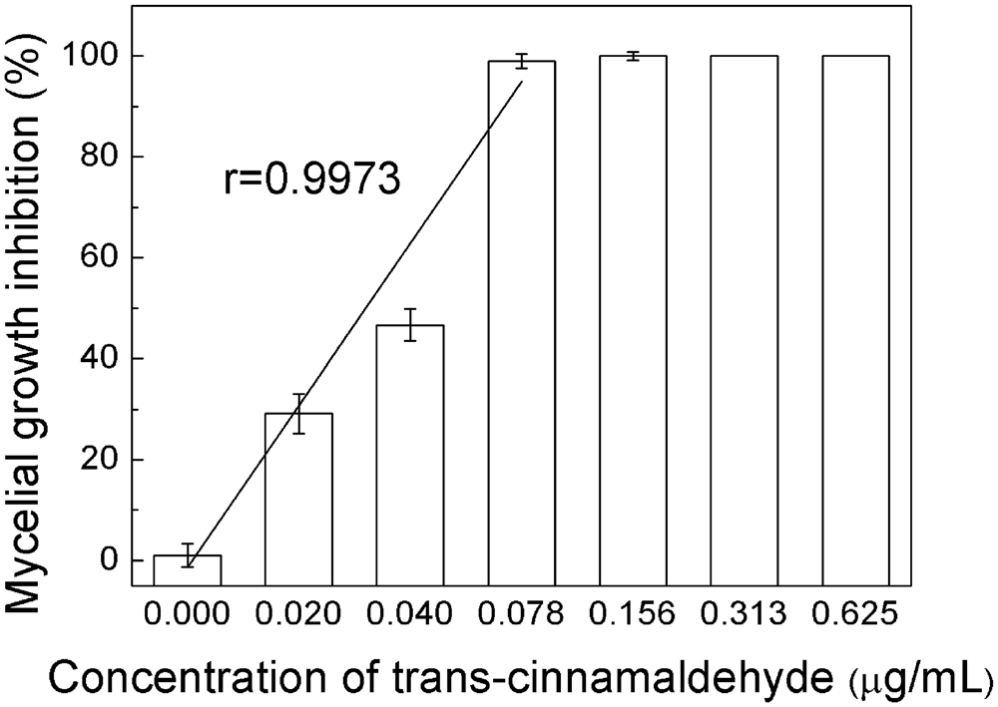
Mycelial growth inhibition of *P. italicum* under different concetration of trans-cinnamaldehyde.

Microtitre Plate Well Assay proved that the lowest concentration of trans-cinnamaldehyde that completely inhibited the growth of the fungus was 0.313 μg/mL which was lower than that of other essential oils, showing in Table 1. Thus, the minimum inhibitory concentration (MIC) of trans-cinnamaldehyde was 0.313 μg/mL. The mycelial growth of *P. italicum* was strongly inhibited by trans-cinnamaldehyde in a dose-dependent manner (r = 0.9973) as shown in Fig. 1. Why the phenomenon that the mycelium growth had been inhibited nearly to 100% under 0.078 μg/mL concentration of trans-cinnamaldehyde which was lower than MIC occurred is that different rate of spore suspension and PDB culture, which was determined by the method system and cannot be avoidable. Low magnitude of MIC made trans-cinnamaldehyde an excellent fungistat.

**Table 1:** Minimum inhibitory concentration of various essential oils against *P. italicum*.

Name of essential oils	Minimum inhibitory concentration
Salicylic acid	2.590 mg/mL
Cinnamic acid	0.400 mg/mL
Thymol	15.938 mg/mL
Carvacrol	0.625 μg/mL
Eugenol	2.500 μg/mL
Carvone	0.750 μg/mL
Trans-Cinnamaldehyde	0.313 μg/mL
Citral	1.250 μg/mL
Geraniol	3.750 μg/mL
Hexanal	10.000 μg/mL

### Effect of trans-cinnamaldehyde on plasma membrane integrity of *P. italicum* spores

**Figure 2:**
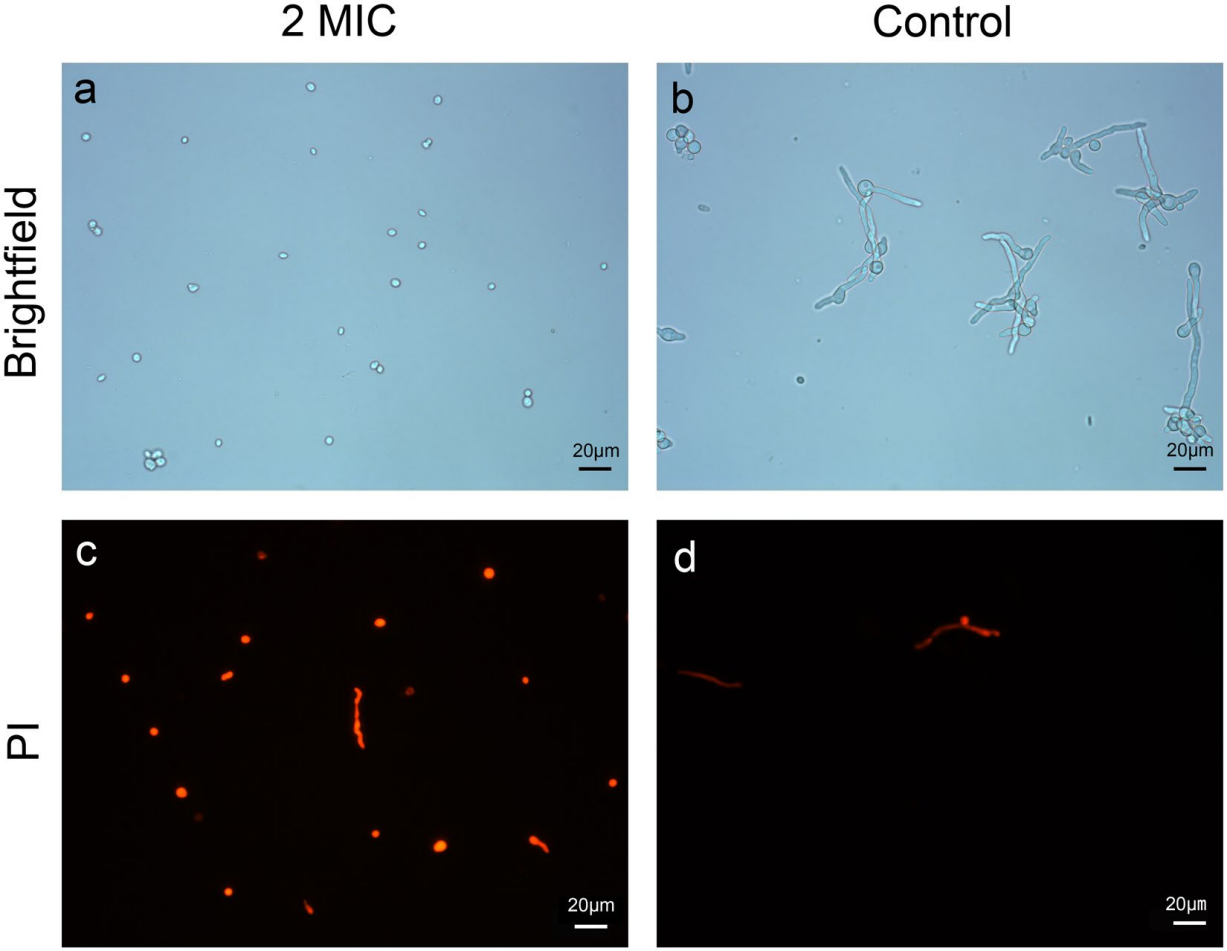
Optical microscope images of *P. italicum* spores after 6 h treatment with 2 MIC trans-cinnamaldehyde.

To test the effect of trans-cinnamaldehyde on plasma membrane integrity, cells were dyed with propidium iodide. And results were shown in Fig. 2. The conidial spores had almost been stained after 6 h treatment with 2MIC trans-cinnamaldehyde, as shown in Fig. 2c. However, when contrasted the cells in bright field and dark field (Fig. 2b,d), it was found that nearly no cells were stained in control group. When observing the cells in bright field (Fig. 2a,b), it was easy to discover that the conidial spore germination had been inhibited to a great extent, which was corresponding to the antifungal assays.

**Figure 3:**
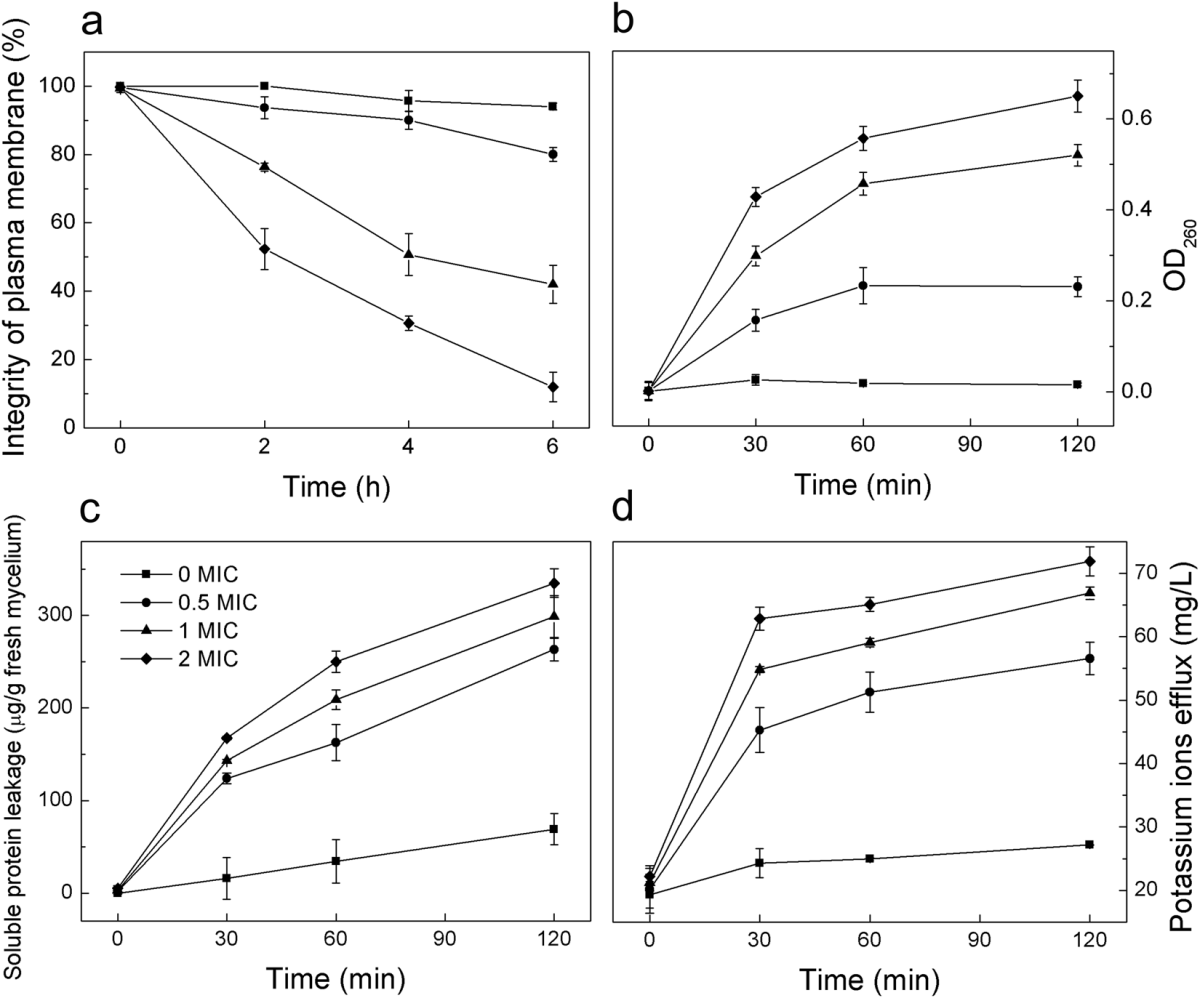
Tendency chart of some index. (**a**) plasma membrane integrity of *P. italicum* spores. (**b**) OD_260_ value of constituents absorbing at 260 nm. (**c**) Soluble protein leakage. (**d**) Potassium ions efflux.

MI of *P. italicum* spores under different dose of trans-cinnamaldehyde were elaborated in Fig. 3. According to Fig. 3a, MI of 2 MIC-treated *P. italicum* was 12% while that of control group was 94% after 6 h incubation. MI of *P. italicum* spores declined with the increase of incubation time in PDB containing different amount trans-cinnamaldehyde. Plasma membrane of *P. italicum* was obviously damaged in PDB with high level trans-cinnamaldehyde. Inversely, MI stayed at high level in control group without trans-cinnamaldehyde, indicating plasma membrane integrity remained complete. The action mode by which trans-cinnamaldehyde interfere the plasma membrane may be related to its dissolution into the hydrophobic domain of the cytoplasm membrane, between the lipid acyl chains, disintegrated the outer membrane of *P. italicum* and finally leading to cell death^27^.

### Release of constituents absorbing at 260 nm and soluble protein

Intracellular materials including nucleic acid which were absorbed at 260 nm in the suspensions as well as soluble protein plays an important role in cell live. Optical density at 260 nm (OD_260_) were used to evaluate the leakage of nucleic acid. The release of constituents absorbing at 260 nm after *P. italicum* was treated with trans-ciannamaldehyde were shown in Fig. 3b. The value of OD_260_ in test group at 30 min were significantly different from what at 0 min (P < 0.05), while the value of OD_260_ in control group did not show any variation. With the treating time went by, the release of cell constituents increased. It was reported that the releasing of cell materials absorbed at 260 nm led to disruption of the cytoplasmic membrane properties^28^. Soluble protein leakage of mycelium cell was shown in Fig. 3c. Soluble protein in suspension increased with the incubation time in all group, but the test groups increased more drastically. The present study of constituents absorbing at 260 nm and soluble protein leaking from cells confirmed that trans-cinnamaldehyde disrupted the cytoplasmic membrane of *P. italicum*, and then led to cell death.

### Efflux of potassium ions

Potassium ions efflux involved in permeability barrier of cytoplasmic membrane^29^. However, maintaining ion homeostasis is integral to the maintenance of the energy status of the cell as well as membrane-coupled, energy-dependent processes such as solute transport, regulation of metabolism, control of turgor pressure and motility^30^. Even relatively slight changes to the structural integrity of cell membranes can detrimentally affect cell metabolism and lead to cell death^26^. In this research, potassium ions efflux in test group were significantly higher than control group after 30 min treatment with trans-cinnamaldehyde (P < 0.05) as shown in Fig. 3d. High level of Potassium ions efflux indicated that trans-cinnamaldehyde severely damaged the plasma membrane of *P. italicum*.

### Accumulation of reactive oxygen species

**Figure 4:**
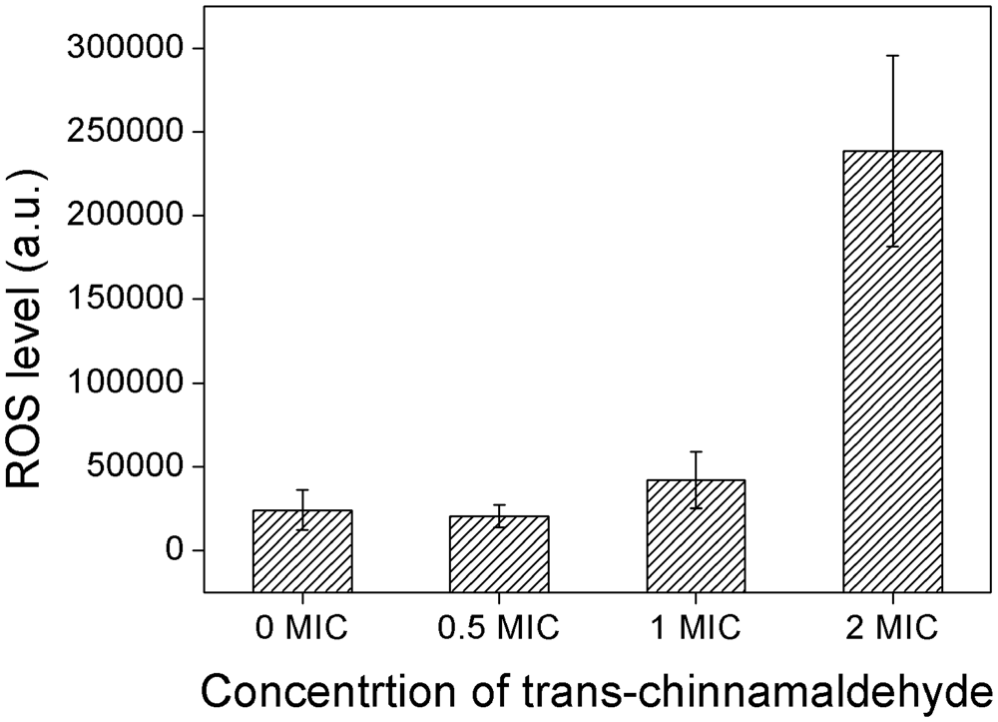
Level of ROS accumulating in mycelium after treating with different concetration of trans-cinnamaldehyde.

In order to assess whether the membrane of mitochondria has been damaged after trans-cinnamaldehyde treatment, level of ROS was determined. As shown in Fig. 4, level of ROS in 2 MIC treated group was significantly higher than other groups, which indicated that massive accumulation of ROS happened. It is widely accepted that massive ROS affects mitochondrial function such as oxidative phosphorylation and mitochondrial membrane permeability. In this case, trans-cinnamaldehyde treatment triggered the production of ROS and thus it accumulated, being a direct danger to mitochondria membrane.

### Ultrastructure change of mycelium after treatment

**Figure 5:**
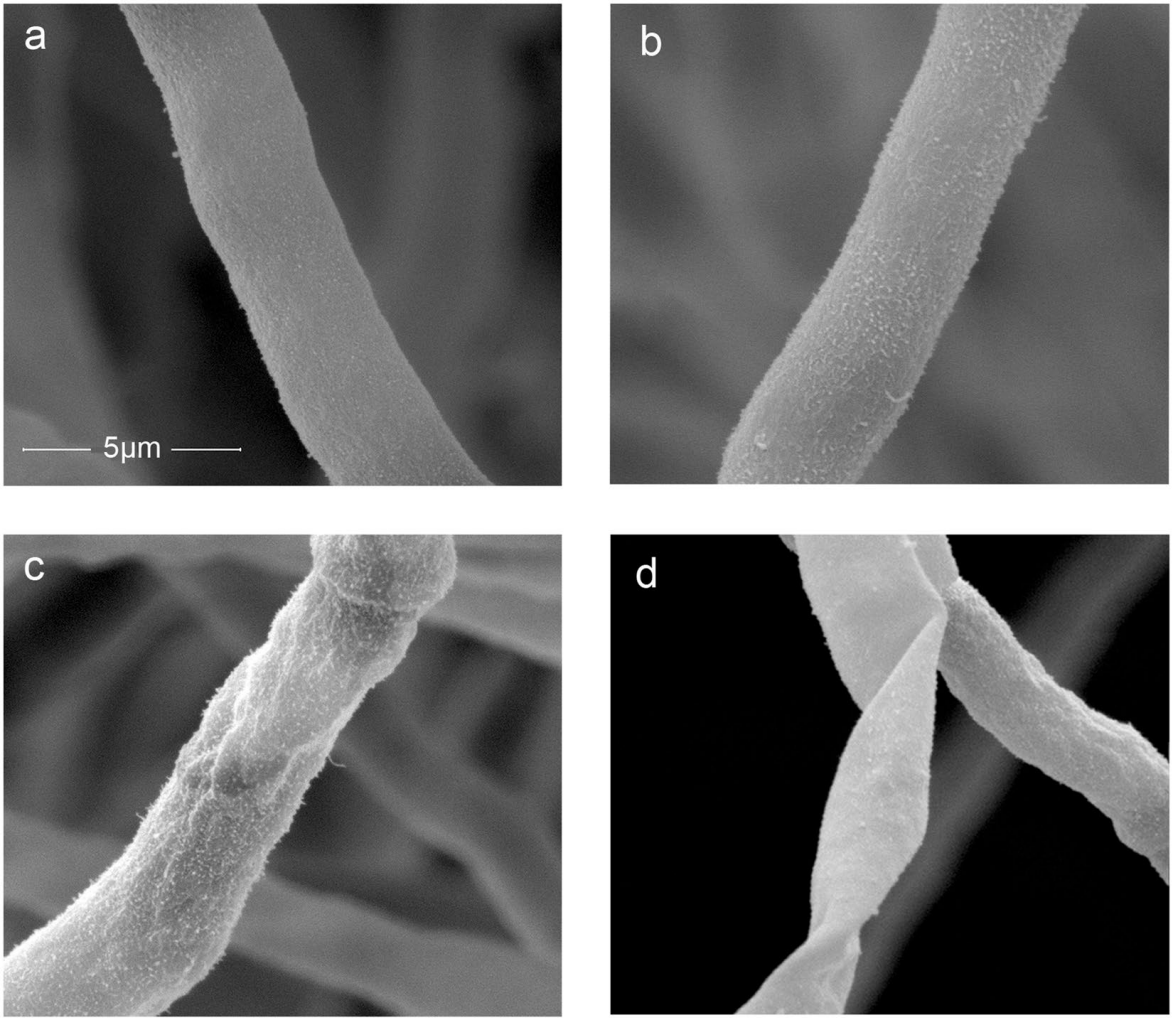
SEM images of mycelium treated under different concentrations. (**a**) control group. (**b**) 0.5 MIC treated group. (**c**) 1 MCI treated group. (**d**) 2 MIC treated group.

SEM is a strong approach to study the ultrastructure of cells. The images of mycelium after various concentration of trans-cinnamaldehyde treatment had been presented in Fig. 5. The control (Fig. 5a) and low concentration group (Fig. 5b) showed a smooth appearance and straight body. It is obvious that the surface of mycelium had been sunken in Fig. 5c,d (group treated with 1 MIC and 2 MIC, respectively). Moreover, the group treated with 2 MIC presented a worse status where the surface was not only sunken but also distorted, suggesting that the structure of cell wall had been disordered so that the skeleton cannot support the cell.

### SERS study on extracellular material

**Figure 6:**
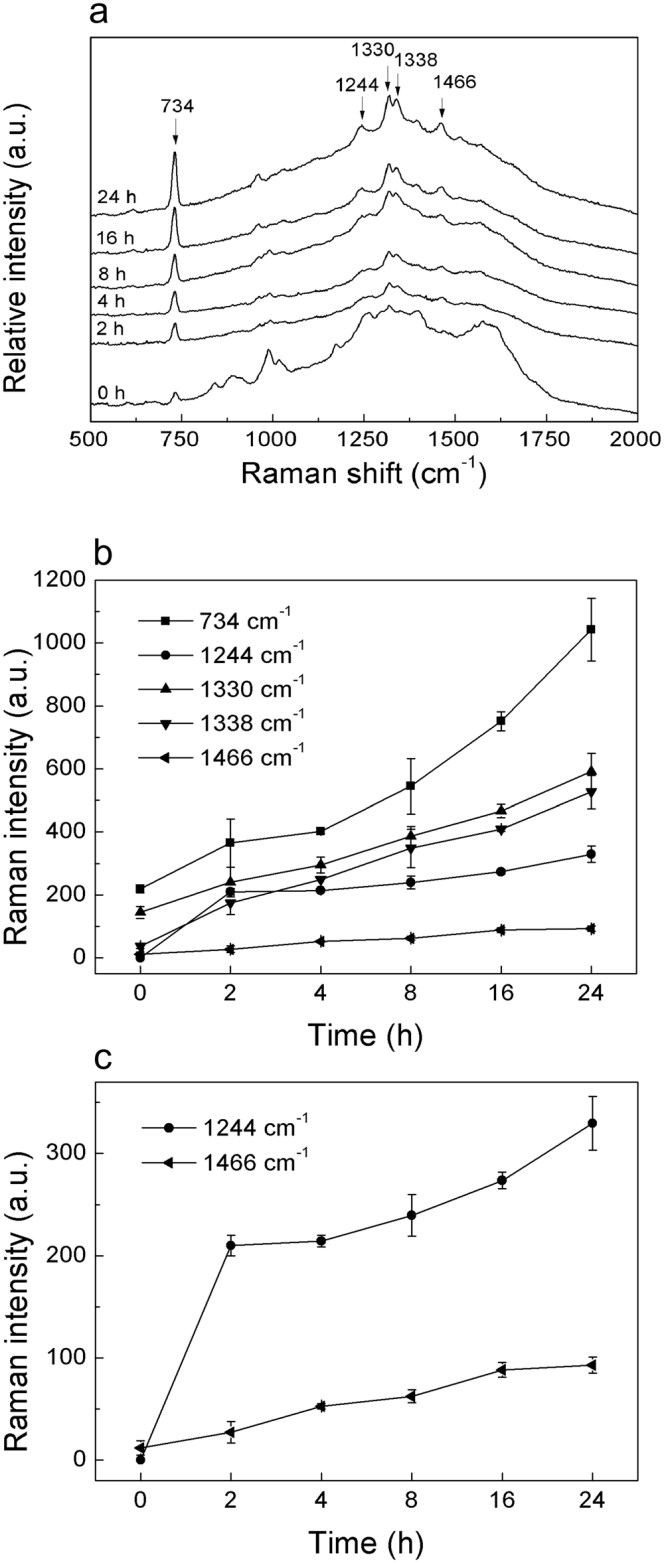
SERS spectra and extracted dataset. (**a**) SERS spectra of extracellular material after treatment with 2 MIC trans*-*cannamaldehyde. (**b**) Trend of peak intensity at different Raman shift. (**c**) Magnified curve of Raman intensity at 1244 cm^−1^ and 1466 cm^−1^.

SERS provided ultrasensitive and fingerprint information of extracellular material after *P. italicum* treated with trans-cinnamaldehyde. Several typical strong SERS peaks were observed as shown in Fig. 6. Intensity at 734, 1330, 1338 cm^−1^ were assigned to nucleic acid. And Intensity at 1244 cm^−1^ was assigned to protein. In addition, intensity at 1466 cm^−1^ was assigned to lipid. According to Fig. 6a, the Raman intensity at 734 cm^−1^ increased dramatically along with the incubation time. Simultaneously, the Raman intensity at 1244, 1330, 1338 and 1466 cm^−1^ went up slowly relative to the baseline. All the detail trend of Raman intensity were elaborated in Fig. 6b,c, expressed as the intensity of individual peak.

**Figure 7:**
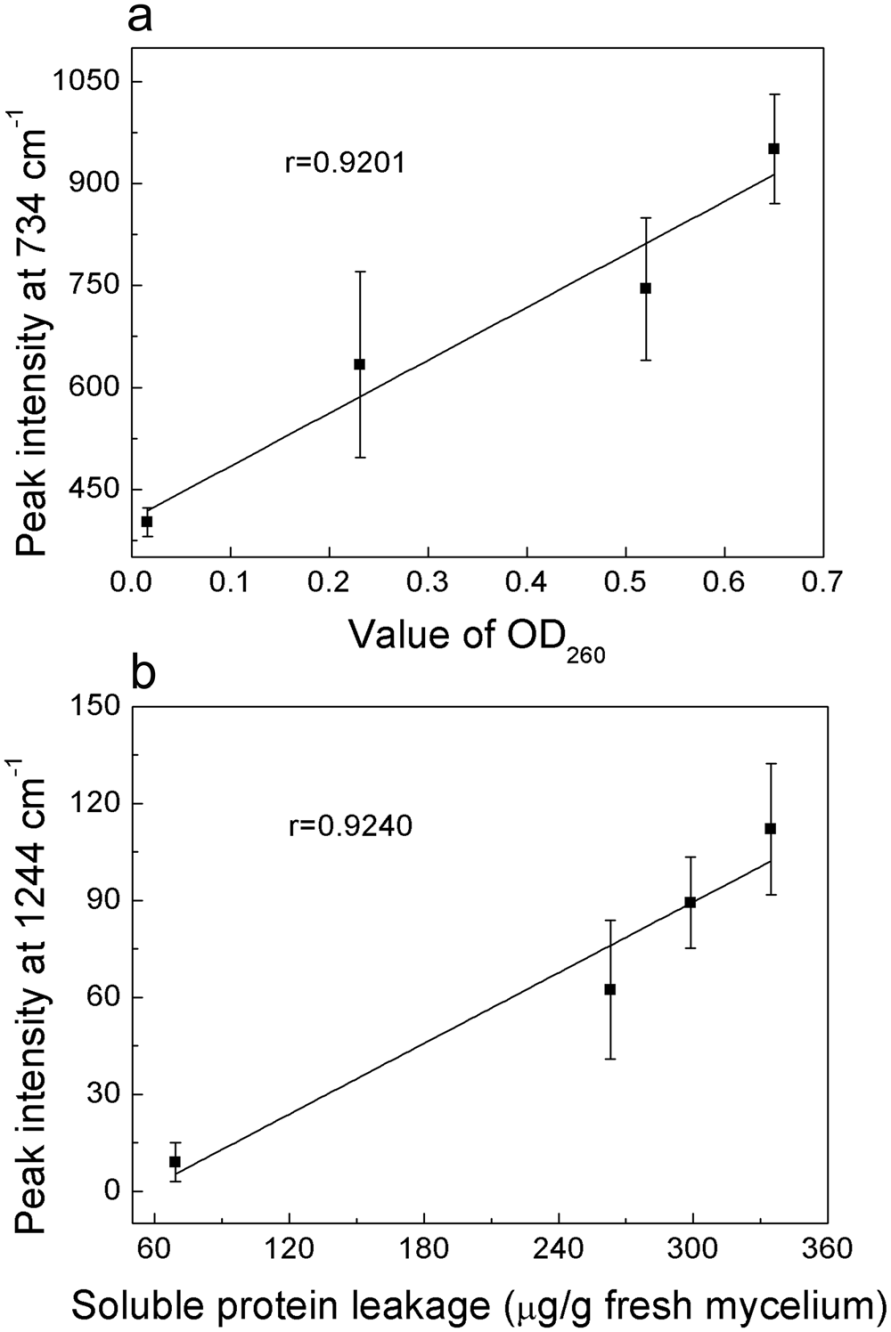
Correlation between Raman intensity and index from previous study. (**a**) The correlation curve between Raman intensity at 734 cm^−1^ and OD_260_ value after 2 h treatment of 2 MIC trans-cinnamaldehyde. (**b**) The correlation curve between Raman intensity at 1244 cm^−1^ and soluble protein leakage after 2 h treatment of 2 MIC trans-cinnamaldehyde.

Intensity at 734 cm^−1^ was fund in adenine, adenosine or adenosine monophosphate (AMP) in previous study^17,31^, which mean 734 cm^−1^ band was assigned to adenine. Further, it comes from adenine breathing mode^31^, precisely. The increase of the intensity at 734 cm^−1^ indicated that the concentration of adenine, adenosine or AMP in suspension raised, which mean that the permeability of plasma membrane had been changed, intracellular material like adenine, adenosine or AMP were able to go through the phospholipid bilayer. This result showed a strong correlation with the OD_260_ value which represented the nucleic acid, with a correlation coefficient of 0.9201 as shown in Fig. 7a. In addition, 1330 and 1338 cm^−1^ were assigned to nucleic acids^32^, the increase of their intensity indicated that the DNA or RNA had gone through the plasma membrane. Moreover, in SERS spectra of DNA, the 734 cm^−1^ band appears stronger for an open double strand due to better contact between the bases and the colloidal gold or silver compared to native DNA^31^. The appearance of the sharp and strong adenine ring breathing peak, after treated with trans-cinnamaldehyde, therefore indicates denaturation of the DNA of *P. italicum*.

Intensity at 1244 cm^−1^ was assigned to Amide-III of protein according to previous study^32,33^. Peak at 1244 cm^−1^ went up gradually after 4 h treatment of trans-cinnamaldehyde as shown in Fig. 6a, which means the ascent of concentration of soluble protein in suspension. Here, it was similar to the soluble protein leakage test, implying that the plasma membrane was disordered. This result was intensively relative to the soluble protein leakage, with a correlation coefficient of 0.9240 shown in Fig. 7b.

Intensity at 1466 cm^−1^ was from lipid^32,34^. Lipid is the main constituent of phospholipid bilayer, keeping the integrity of cell membrane so as to serve important functions in maintaining fungal viability^25^. The increase of intensity at 1466 cm^−1^ in suspension indicated that trans-cinnamaldehyde destroyed the phospholipid bilayer of mycelium of *P. italicum*, finally, led to cell death.

## Conclusion

To learn the mechanism of trans-cinnamaldehyde inhibiting *P. italicum*, to control the diseases of blue mold of citrus fruits and prevent economic losses. In this paper, we revealed the mechanism of trans-cinnamaldehyde inhibiting *P. italicum*, via membrane damage using SERS which offered ultrasensitive and fingerprint information. Membrane integrity test, Potassium ions efflux and membrane permeability tests indicated that the plasma membrane of *P. italicum* had been severely destroyed by trans-cinnamaldehyde and the cell wall collapsed referring to SEM images. Determination of ROS implied that mitochondria membrane would be disordered. SERS of supernatant suggested several Raman intensity increased, furthermore, peaks at 734, 1330 and 1338 cm^−1^ were related to nuclei acid release, peak at 1244 cm^−1^ was related to protein leakage, and peak at 1466 cm^−1^ was related to lipid losses. The Raman intensity has shown a strong correlation with either OD_260_ value or soluble protein leakage, which mean SERS would be an alternative to analyze the antifungal mechanism of essential oil on fungus. All proves pointed to the damage of plasma membrane of *P. italicum* caused by trans-cinnamaldehyde. The future study will focus on the application of SERS on *in situ* detection of antifungal mechanism in living fungus.
